# Repeatability, Reproducibility, and Concurrent Validity of a Stethoscope and Health App System for the Quantification of Breath Rate in Healthy Adults: Repeatability and Validity Study

**DOI:** 10.2196/41845

**Published:** 2023-01-12

**Authors:** Ricardo Becerro de Bengoa Vallejo, Marta Elena Losa Iglesias, Oscar David Robles Sanchez

**Affiliations:** 1 Departamento de Enfermería Facultad de Enfermería, Fisioterapia y Podología Universidad Complutense de Madrid Madrid Spain; 2 Departamento de Enfermería y Estomatología Facultad de CC de la Salud Universidad Rey Juan Carlos Alcorcón Spain; 3 Departamento de Ciencias de la Computación, Arquitectura de Computadores Lenguajes y Sistemas Informáticos y Estadística e Investigación Operativa, Esc Tec Sup de Ingeniería Informática Universidad Rey Juan Carlos Mostoles Spain

**Keywords:** breath rate, stethoscope, smartphone app, breathing rate, vital sign, respiration, mobile phone app, health app, mobile app, mHealth, mobile health, measurement, breathing, assessment, monitoring, reliability, validity, medical device, medical instrument

## Abstract

**Background:**

Apps for smartphones that can measure the breathing rate easily can be downloaded.

**Objective:**

The aim of this study was to demonstrate agreement in measuring breath rates between the stethoscope and Breath Counter health app.

**Methods:**

We performed a repeatability study with 56 healthy volunteers. The patient’s demographic data and breathing rates per minute were collected. Breathing rates were measured via two methods: (1) using a stethoscope placed in the upper area of the right lung and (2) a Breath Counter app developed by Vadion on a Samsung Fold smartphone.

**Results:**

This study demonstrated high repeatability and validity with respect to the breathing rate parameter of healthy adults using the aforementioned 2 systems. Intrasession repeatability measure using the intraclass correlation coefficient was >0.962, indicating excellent repeatability. Moreover, the intraclass correlation coefficient between methods was 0.793, indicating good repeatability, and coefficients of variation of method errors values were 1.83% with very low values in terms of other repeatability parameters. We found significant correlation coefficients and no systematic differences between the app and stethoscope methods.

**Conclusions:**

The app method may be attractive to individuals who require repeatability in a recreational setting.

## Introduction

In recent years, the need for health promotion programs across the general healthy population has increased [1], and mobile app programs have been used to prevent and manage risk factors, increase physical activity, improve dietary habits [2], promote weight loss, and reduce smoking, stress, depression, and obesity [3]. Often, individuals report having difficulty accessing health promotion programs, including advice, information, feedback, and self-monitoring, given the fast pace of modern life; hence, mobile app programs could provide an alternative [4]. For example, monitoring of breath is important for the management of fatigue in physical performance in healthy people [5].

Hence, breath rate is usually measured by a health care professional with an instrument called a stethoscope [6], but due to the rise in self-care and the lack of health care resources, society is looking for tools that are easy to use and within reach and understanding of the general population. In this sense, the Breath Counter app by Vadion [7] has been developed and measures this breathing rate.

Since the app offers little information regarding its effectiveness, the aim of this study was to assess the repeatability and reproducibility of this type of smartphone app using an Android-based operating system when compared with the conventional stethoscope to guide effective use by the general population. Based on the increased use of health-related apps, we hypothesized that no systematic differences between the app and stethoscope measurements would be detected. Our main goal was to demonstrate agreement in measuring breath rates between the stethoscope and Breath Counter health app methods.

## Methods

### Participants and Methods

We performed a repeatability study with healthy volunteers from June to July 2022. Healthy subjects from university staff and students volunteered to participate.

### Participants

The selection and inclusion criteria were being older than 18 years (legal age) and free from any cardiovascular, neurological, respiratory, or musculoskeletal diseases. The exclusion criteria considered several parameters: (1) refusal to provide informed consent, (2) other injuries that may generate fear of movement, and (3) inability to understand and carry out study instructions.

The participants’ demographic data and breathing rate per minute were collected. Breathing rate was measured with two methods: (1) a stethoscope placed in the upper area of the right lung [8] and (2) the Breath Counter app developed by Vadion on a Samsung Fold smartphone with the Android 12 operating system under One UI 4.1 [9]; the app can also be used in smartphones using the iOS system. The smartphone, with the Breath Counter app opened, is placed on the abdomen without a case or accessories, and from that position, the measurement will be collected for 1 minute. At the same time, the stethoscope was placed on the chest of the participant to avoid any spontaneous fluctuations in breath rate. The breath sounds are heard best over the first and second intercostal spaces beside the sternum on the anterior side of the chest. These sounds are produced when air moves through the lungs’ large airways and has shown its validity [10,11].

To avoid breathing rate variations, participants remained lying on a stretcher for 10 minutes prior to the measurements, and breath measurements were taken 3 consecutive times with each method. The same operator performed both methods in a randomized order, using the same equipment.

### Sample Size Calculation

Sample size calculation was performed on the basis of the correlation between 2 independent groups using the G*Power 3.1.9.2 software, a 2-tailed hypothesis, an effect size of 0.40, an α error probability of .05 with a β level of 20%, and the desired power analysis of 80% (1-β error probability). Therefore, a total sample size of at least 44 participants was calculated.

### Statistical Analysis

Regarding quantitative data, all variables were examined for normality of distribution using the Kolmogorov-Smirnov test, and data were considered normally distributed if *P*>.05.

Descriptive statistical analyses are presented as mean (SD) and median with its 95% CI of 3 measurements. The Mann-Whitney *U* test for independent samples was used to determine systematic differences between the breathing rate values obtained using the 2 systems.

Intratrial repeatability was established using the 3 measurements with both methods during one session. Intraclass correlation coefficients (ICC) using the (1,1) model were calculated to determine repeatability between trials when using each system, and ICC values of <0.5 were considered indicative of poor repeatability, values between 0.5 and 0.75 indicated moderate repeatability, values between 0.75 and 0.9 indicated good repeatability, and values > 0.90 indicated excellent repeatability [12].

The standard error of the mean (SEM) was calculated from the ICCs and SDs for each of the 3 measurements. SEM was calculated with the following formula: SD × square root (1 − ICC) [13,14].

The coefficient of variation (CV) and the percent error were calculated for intrasession repeatability. The CV is calculated as the mean normalized to the SD. This value represents the amount of variation between trials, normalized to the mean for each variable. A higher coefficient of variation shows greater heterogeneity of variable values, and a lower coefficient of variation indicates greater homogeneity in the values of the variable. Similarly, the percent error is calculated as the SEM divided by the mean per 100 and provides an estimate of the inherent error or variability normalized to the mean.

In concordance, the results of breathing rate measurements using the 2 methods were compared using the ICC [7]. Concurrent validity between the 2 systems, the Breath Counter app and stethoscope methods, were calculated using ICCs [15].

Coefficients of variation of method errors (CVME) and 95% limits of agreement (LoA) were also calculated for the absolute comparison of parameters. As shown in the formula below, CVME values were converted into percentages by calculating the CVME obtained using the SD of differences between the results obtained using the 2 systems. CVME expresses the differences between values obtained using the 2 systems as a percentage and, in doing so, CVME can be used as a clinically useful indicator of consistency, since it is unaffected by sample heterogeneity [12]:

ME = SD / √2

CVME = 2ME / (X1+X2) × 100%

Bland-Altman analysis was used to determine the LoA [16] between the 2 measurement methods. Bland-Altman analysis quantifies the amount of agreement between 2 methods of measurement by constructing LoA. These limits are calculated by using the mean and SD values of the differences between 2 measurements.

On Bland-Altman analysis, the LoA are defined as 95%, as the authors recommended that 95% of data points should lie within 2 SDs of the mean difference. The results of this analysis are conventionally displayed graphically using a scatter plot, in which the Y axis shows the difference between 2 paired measurements, and the X axis represents the average of these measurements.

Repeatability coefficients (RCs) were used to evaluate the level of agreement between the Breath Counter app and stethoscope methods.

The RC was calculated in accordance with Bland and Altman [13] as 1.96 times the SD of the differences between the paired measurements. The difference between the 2 measurement systems is expected to be less than this coefficient at a probability of 95%.

Pearson correlation and linear regression analyses were also performed. The Pearson correlation coefficient (*r*) was used to measure the strength of association between methods of measurement. The correlation values are considered to indicate a good correlation at *r*=0.41-0.60, a very good correlation at *r*=0.61-0.80, and an excellent correlation when *r*>0.81 [12].

Linear regression analysis was used to predict the breathing rate values from each system. Finally, we produced Bland-Altman plots [13] to display the agreement between the 2 devices. These plots show the difference between each pair of measurements on the y-axis against the mean of each pair of measurements on the x-axis.

These statistical methods are generally accepted for evaluating the agreement of 2 systems of clinical measurements irrespective of the distribution of variables and residuals [12]. A *P* value of <.05 was considered significant (SPSS for Windows, version 20.0; SPSS Inc).

### Ethical Considerations

The ethics committee of Universidad Rey Juan Carlos, Spain (code 0106202216022), approved this research, and all subjects signed the informed consent form prior to the beginning of the study. Finally, the Helsinki declaration and all human experimentation guidelines were respected [16].

## Results

All variables showed a normal distribution (*P*>.05), except the stethoscope rate (*P*=.001). A total of 56 participants participated in the study (28 males and 28 females) and their characteristics are shown in Table 1.

The intrasession repeatability data, represented by the ICC, SEM, CV, and percent error, and normative data represented by mean (SD) and median and 95% CI values for the variable breathing rate repeatability trials using the 2 methods, are presented in Table 2. The results of the trial’s intrasession repeatability produced an excellent ICC, low SEM, low percent errors, and low CVs. The results represent a small error that may occur within trials when using any of the methods tested.

The median and 95% CI values of the variable using the 2 methods are presented in Table 3. The Mann-Whitney *U* test was used to determine systematic differences between the breathing rate values obtained with the 2 methods, and we determined that values were similar when comparing the Breath Counter app and stethoscope methods with no significant differences between the 2 methods. Concurrent validity between both methods was calculated using ICCs [1,2] resulting in ICC values that were considered in the “good repeatability” range. Correlation analysis between methods showed a “good correlation.” Other validity parameters, such as LoA, CV%, CVME, and RC, were very small and showed excellent concurrent validity.

**Figure 1:**
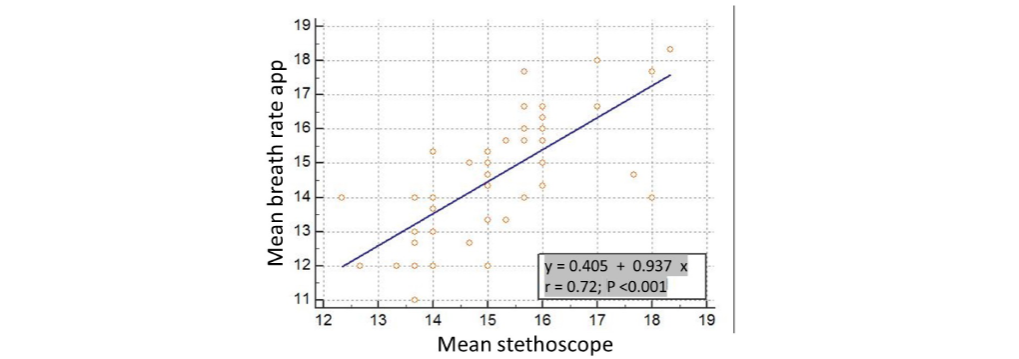
Linear regression graph and mathematical formula for the Breath Counter app and stethoscope methods.

We used linear regression analyses to evaluate the relationship between the Breath Counter app and stethoscope methods. The app data had a significant positive correlation with the stethoscope (*R*²=0.521, *P*<.001), as shown in Figure 1, with a regression equation of y = 0.405 + 0.937x. The significant association suggests that a linear regression model is optimal for prediction using the app approach.

**Figure 2:**
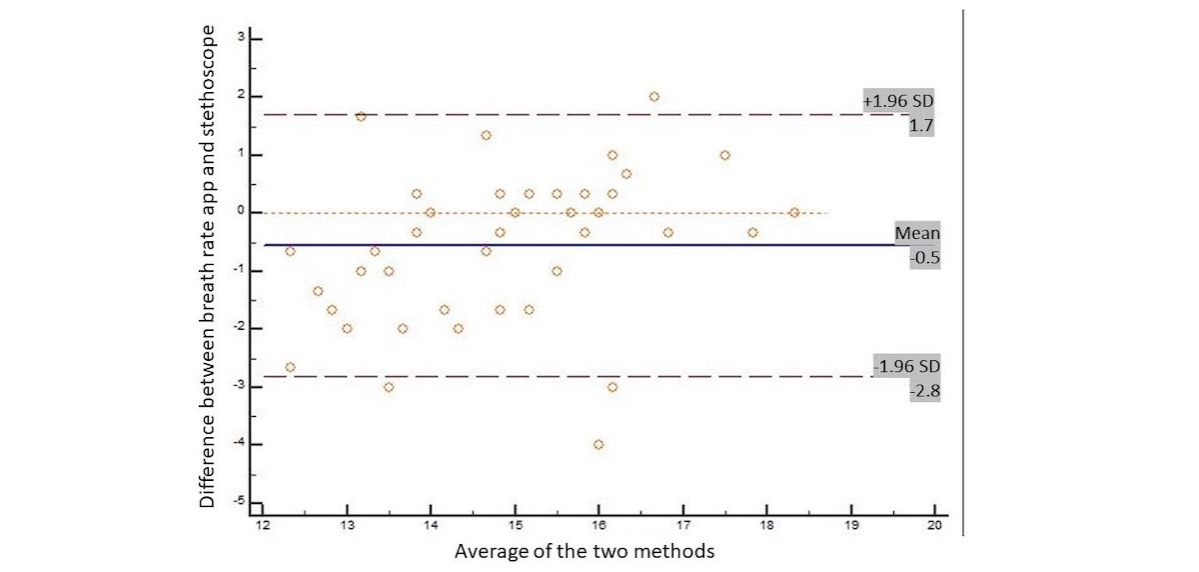
Bland-Altman plot comparing the Breath Counter app and the stethoscope methods for determining the breath rate per minute (bpm) for individual participants.

Figure 2 displays the Bland-Altman plots for the breathing rate using both methods. For each variable and for almost every participant, the difference between the means of the methods fell within the 95% CI of all measurements.

**Table 1:** Descriptive data of the participants.

Descriptive Data	Total group (n=56), mean (SD; 95% CI)	Females (n=28), mean (SD; 95% CI)	Males (n=28), mean (SD; 95% CI)	*P* value
Age (years)	35.90 (9.18; 33.44-38.37)	35.53 (8.65; 32.18-38.89)	56.29 (9.86; 32.47-40.12)	.76^a^
Weight (kg)	66.41 (12.70; 63.01-69.82)	58.42 (11.53; 53.95-62.90)	74.70 (7.55; 71.77-77.63)	<.001^a^
Height (m)	170.56 (8.05; 168.40-171.72)	165.00 (5.49; 162.86-167.13)	176.33 (5.95; 174.02-178.64)	<.001^a^
BMI (kg/m^2^)	22.54 (2.88; 21.77-23.31)	21.35 (3.23; 20.10-22.61)	23.77 (1.81; 23.07-24.48)	.001^a^

^a^A Student *t* test for independent samples was performed. In all analyses, *P*<.05 (with a 95% CI) was considered significant.

**Table 2:** Intrasession repeatability of breathing rate (per minute) measurements using different methods.

Method	Mean (SD; 95% CI)	Median (95% CI)	Intraclass correlation coefficient (95% CI)	SE of the mean	Coefficient of variation	Percent error, %
Breath Counter app	14.65 (1.66; 14.20-15.10)	14.66 (14.25-15.07)	0.962 (0.940-0.976)	0.32	0.113	2.18
Stethoscope	15.20 (1.28; 14.85-15.54)	15 (15.00-15.66)	0.952 (0.925-0.970)	0.28	0.084	1.84

**Table 3:** Mean (SD) values for breathing rate and concurrent validity measured with the Breath Counter app and stethoscope methods.

Stethoscope method, median (95% CI)	App method, median (95% CI)	Intraclass correlation coefficient (95% CI)	*P* value^a^	Pearson *r* (*P* value)	*R*²	Limits of agreement (95% CI)	Coefficient of variation, %	Coefficients of variation of method errors	Repeatability coefficient	Repeatability coefficient, %
15 (15.00 to 15.66)	14.66 (14.25 to 15.07)	0.793 (0.59 to 0.887)	.08	0.722 (<.001)	0.521 (<.001)	−0.5 (−2.80 to 1.70)	2.58	1.83	0.48	1.90

^a^A Mann-Whitney *U* test for independent samples was performed. In all analyses, *P*<.05 (with a 95% CI) was considered significant.

## Discussion

### Principal Findings

This study was conducted to investigate the intrasession repeatability and concurrent validity between the clinical standard breathing rate per minute measured using a stethoscope and the Breath Counter app among healthy young adults. This type of technology would be useful in certain situations or in patient populations in whom it is difficult to measure breathing rates with traditional methods, such as using a stethoscope. For example, in athletes, it can be difficult to measure breathing rates because of their high frequency or lack of a stethoscope. However, with the use of the breath counter app, one can assess breathing rates and monitor performance. In a home setting, it could be difficult for an older person to use a stethoscope; hence, using the Breath Counter app could be beneficial in assessing breathing rate and potential influences from stress or nervousness or any disease or sequela due to COVID-19.

In our study, we examined a healthy adult population. It is essential that the validity of breathing rate measurement systems is established in the populations for their intended use. The Breath Counter app system provided consistent intrasession results between trials with a very low intrasession variability with an ICC of 0.972, and nearly all of the percent errors were below 2.188%.

These findings suggest that breathing rate measurements with an app are appropriate for use in evaluating differences between participating groups. The SEM and percent error values are important variables that should be considered when formulating research protocols that use the low-cost breathing rate method based on a smartphone app. The sample size to determine significant changes can be based directly on these measures of intrasession repeatability.

The ICCs are a mathematical determination of the replication between multiple numerical sets and are commonly used for scientific measurements to represent the repeatability of the measurement [15,17]. It has been suggested that ICCs of >0.75 indicate good repeatability [15].

Although ICCs provide a numeric value for the repeatability of a measurement device, they do not describe the amount of error or inherent variability that is expected each time the measurement is performed. Assessing the error or variation each time a trial is performed is extremely important when capturing physiological data for which small differences between trials are expected. The SEM is another mathematical formula that uses the ICC and SD values to calculate the amount of expected error for the measurement device or individual [15].

The SEMs and percent errors for the breathing rate variable in this study were very low, suggesting that the variables are acceptable to use when assessing change before and after the intervention, or when measuring differences between participant groups. Absolute repeatability is as important as relative repeatability. SEM is a quantitative expression of the range of errors that can occur whenever the same participant repeats certain tests [14]. In this study, the calculated intrasession SEM was very low, indicating strong absolute repeatability. The SEM values provided in this analysis will allow future researchers to make clinical judgments regarding what degree of change is due to factors beyond errors associated with the normal variability of measuring between trials or between sessions.

No systematic differences between the Breath Counter app and stethoscope methods were found, and a high level of correlation was determined between the 2 methods. Menz et al [18] suggested that although ICC is a more appropriate indicator of repeatability than simple correlation coefficients (Pearson *r* and Spearman ρ), a higher ICC does not necessarily ensure a high repeatability. If the values of a sample are distributed over a wide range, a relatively high ICC can be achieved even though score differences between the 2 measurements may be widely distributed. Thus, it has been asserted that both CVs and LoA [13] must be used concurrently to reduce the effects of such intrinsic limitations and to ensure absolute repeatability.

This study demonstrated high repeatability and consistency between methods with respect to the breathing rate parameter of healthy adults using the Breath Counter app and the stethoscope. For all parameters, the ICC was >0.793, indicating good repeatability. Moreover, CVME values for breathing rate parameters were 1.83%, and 95% LoA values, including zero, were within a narrow range with a symmetric distribution. These findings indicate slight changes between repeated measures using 2 methods and systematic bias was rarely observed. We found significant correlation coefficients and no systematic differences between the Breath Counter app and stethoscope methods, and the app’s precision is very high. However, the precision of prediction between both methods was very consistent. The distribution of residuals also indicates a significant variation in the prediction of stethoscope values from samples of Breath Counter app trials. The findings suggest that simple linear models may represent the association between stethoscope and app values appropriately. Accordingly, using the accepted app method, breathing rate may be a reliable proxy.

### Limitations

The fact that breathing rate was evaluated in healthy adults, and not in those with systemic or pulmonary disease, may represent a study limitation. While a specific measure may be valid in a young healthy subject, the same may not be true for an older person with an abnormal breathing rate pattern. Future work with apps should evaluate the repeatability and normative values for various ages or pathologies that are known to be susceptible to measure in high-risk patients, including those with COVID-19 or those with post–COVID-19 condition.

This is the first study of its kind to examine the intrasession repeatability and validity of an app system when compared to that of the conventional stethoscope, and more studies are needed to demonstrate that app systems are valid instruments for the assessment of breathing rate per minute in healthy adults.

### Conclusions

The Breath Counter app showed a strong correlation with the stethoscope, and the app needs to apply the regression formula as a corrective factor to correlate with the breathing rate. The app method may be attractive to individuals who require repeatability in a recreational setting. Such features also make this system a viable option for use in a sports environment.

Our findings suggest that simple linear models may represent the association between stethoscope’s and Breath Counter app’s values appropriately. Accordingly, using the accepted app method, breathing rate may be a reliable proxy. Sports coaches could implement the measurement of breathing rate through an app or by monitoring healthy adults’ progress during sports or training interventions. Therefore, future research should assess individuals with documented systemic disease or lung injuries to determine the suitability and validity of these app in such clinical settings.

## Abbreviations

CV: coefficient of variation
CVME: coefficients of variation of method errors
ICC: intraclass correlation coefficients
LoA: limits of agreement
RC: repeatability coefficient
SEM: standard error of the mean
